# Synthesis of Chiral Macrocyclic or Linear Pyridine Carboxamides from Pyridine-2,6-dicarbonyl Dichloride as Antimicrobial Agents

**DOI:** 10.3390/molecules15096588

**Published:** 2010-09-20

**Authors:** Rashad A. Al-Salahi, Mohamed A. Al-Omar, Abd El-Galil E. Amr

**Affiliations:** Department of Pharmaceutical Chemistry, College of Pharmacy, King Saud University, Riyadh 11451, Saudi Arabia

**Keywords:** synthesis, chiral macrocyclic, pyridine-2,6-dicarbonyl dichloride, antimicrobial activity

## Abstract

A series of chiral linear and macrocyclic bridged pyridines has been prepared starting from pyridine-2,6-dicarbonyl dichloride **(2)**. The coupling of **1** with D-alanyl methyl ester gave 2,6-bis-D-alanyl pyridine methyl ester (**3**). Hydrazinolysis of **3** with hydrazine hydrate afforded bis-hydrazide **4**. The latter was reacted with thiophene-2-carbaldehyde, phthalic anhydride or cyclohexanone to afford bis-carboxamide pyridine derivatives **5-7,** respectively. Compound **4** was coupled with *p-*methoxy- or *p*-nitroaceto-phenone to yield compounds **8** and **9**. In addition, **4** was reacted with 1,2,4,5-benzenetetra-carboxylic acid dianhydride or 1,4,5,8-naphthalenetetracarboxylic acid dianhydride to afford the macrocyclic octacarboxaamide pyridines **10** and **11**. The detailed synthesis, spectroscopic data and antimicrobial screening for the synthesized compounds are reported.

## 1. Introduction

Synthesis of chemical modifications of existing antibacterial agents in order to generate novel molecules with better therapeutic properties is necessary because of the emergence of multidrug resistant bacteria [1]. In continuation of our previous investigations [2,3,4], we have previously reported the synthesis and biological activity screening of some dipicolinic acid bis-L-amino acid hydrazide derivatives [3] and their corresponding acids [5]. Compounds of this kind have attracted considerable attention as inhibitors of ribonucleoside diphosphate reductase [6]. Synthetic macrocyclic peptides are still the subject of intensive research with respect to their therapeutic applications [7], as well as their binding properties [8]. We also demonstrated that some peptido-heterocyclic derivatives exhibit a general ionophoric potency towards divalent cations [9] and are useful for assembling novel thiocyanate-selective membrane sensors [10]. On the other hand, Schiff base and other heterocyclic derivatives were reported to possess diverse biological activities, such as antibacterial [11,12,13,14] and anti-inflammatory [15,16,17] properties. Recently, we have demonstrated the significance of 2,6-di-substituted pyridine derivatives as biologically active congeners [18,19,20,21,22,23]. In view of these observations and as a continuation of our previous work in peptido-heterocyclic chemistry, we have synthesized some new compounds containing amino acid and hetero-organic moieties, and tested them for selected biological activities.

## 2. Results and Discussion

### 2.1. Chemistry

**Scheme 1:**
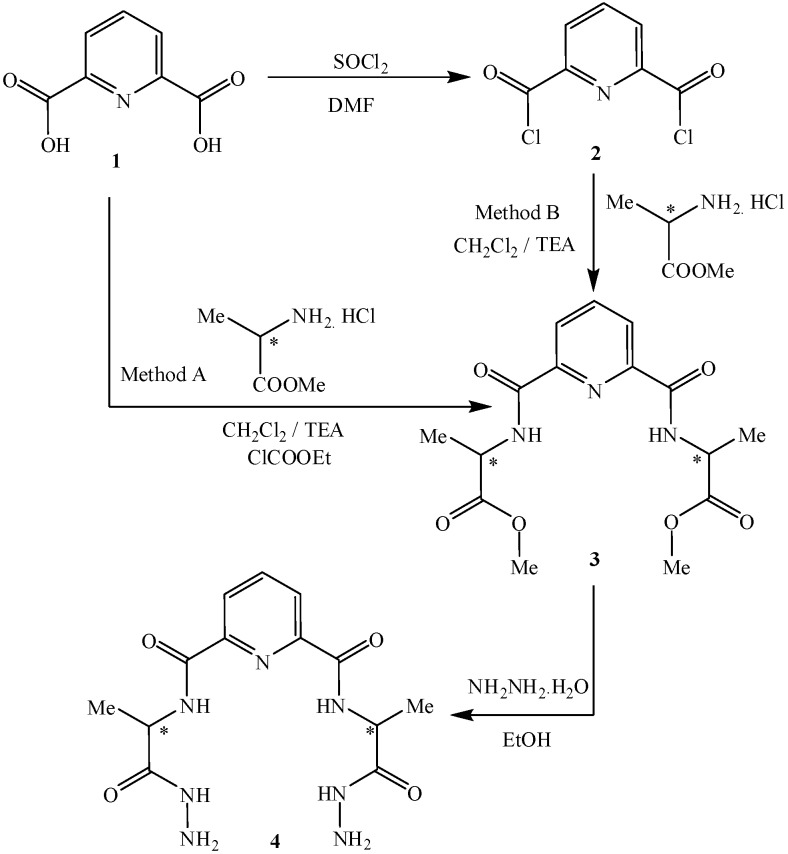
Synthetic routes to compounds **3** and **4**.

D-Alanyl methyl ester was initially coupled with 2,6-dipicolinic acid (**1**) (Mixed Anhydride Method, Method A] [5] or with pyridine-2,6-dicarboyl dichloride (**2**) via the conventional acid chloride method (Methid B) [3] to give the corresponding bis-ester **3**. Hydrazinolysis of **3** with hydrazine hydrate in absolute ethanol afforded the corresponding bis-hydrazide **4** (Scheme 1).

**Scheme 2:**
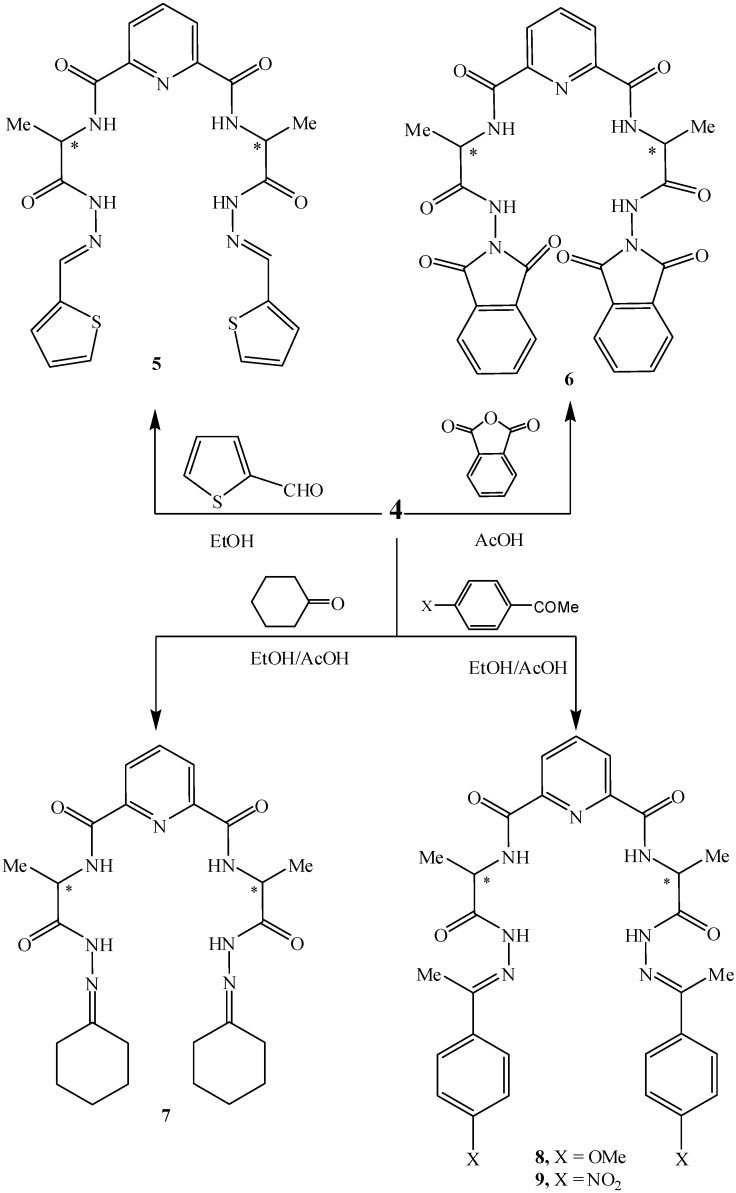
Synthetic routes to compounds **5-9**.

The reaction of bishydrazide **4** with thiophene-2-carbaldehyde in refluxing ethanol afforded the corresponding hydrazone **5,** but when it was reacted with phthalic anhydride in refluxing glacial acid it gave the corresponding imide **6**. In a similar way, treatment of bis-hydrazide **4** with carbonyl compounds, namely, cyclohexanone, *p*-methoxyacetophenone or *p*-nitroacetophenone in refluxing ethanol in the presence of few drops of glacial acetic acid afforded the corresponding bis-(cyclohexyl tetraamide)pyridine derivative **7,** bis-(substituted aryl tetraamide)pyridine derivatives **8** and **9**, respectively (Scheme 2).

**Scheme 3:**
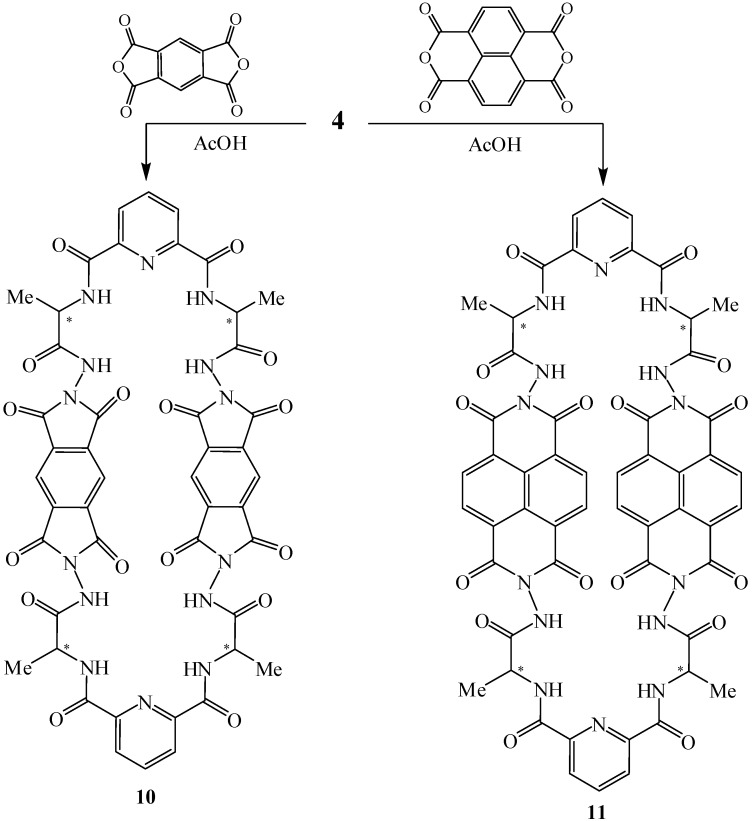
Synthetic routes to compounds **10** and **12**.

Condensation of the same hydrazide **4** with selected tetraacid dianhydrides, namely, 1,2,4,5-benzenetetracarboxylic acid dianhydride or 1,4,5,8-naphthalenetetracarboxylic acid dianhydride in refluxing acetic acid afforded the corresponding macrocyclic octaamide-tetraimide pyridine derivatives **10** and **11**, respectively (Scheme 3).

**Table 1:** Melting points, crystallization solvents, yields, molecular formulae and molecular weights of compounds **3-9**.

Comp. No.	Mp (ºC)	Cryst. Solv.	Yield (%)	[α]^30^_D_ [MeOH]	Molecular Formula (Mol. Wt.)
**3**	148-250	EtOH/diethyl ether	75 [A]; 80 [B]	+ 68	C_15_H_19_N_3_O_6_ (337.13)
**4**	193-195	EtOH	76	+ 45	C_13_H_19_N_7_O_4_ (337.33)
**5**	157-159	EtOH	65	+ 32	C_23_H_23_N_7_O_4_S_2_ (525.60)
**6**	302-304	AcOH/ether	80	+ 8	C_29_H_23_N_7_O_8_ (597.54)
**7**	135-137	EtOH/diethylether	65	+ 18	C_25_H_35_N_7_O_4_ (497.59)
**8**	139-140	EtOH	70	+34	C_31_H_35_N_7_O_6_ (601.65)
**9**	152-154	EtOH	65	+ 54	C_29_H_29_N_9_O_8_ (631.60)
**10**	201-203	DMF/H_2_O	60	+ 24	C_46_H_34_N_14_O_16_ (1038.85)
**11**	232-234	DMF/H_2_O	65	+ 16	C_54_H_38_N_14_O_16_ (1138.96)

The structures of newly synthesized compounds **3-11** were confirmed by IR, ^1^H-NMR, ^13^C-NMR and mass spectra.

### 2.2. Antimicrobial Testing

Preliminary biological activity screening of the synthesized compounds at 50 μg/mL has been performed against microorganisms representing Gram-positive bacteria (*Bacillus subtilis* and *Staphylococcus aureus*), Gram-negative bacteria (*Escherichia coli*) and fungi (*Candida albicans*), using the bioassay technique of antibiotics [24] specified in US Pharmacopeia. From Table 2, it appears that the synthesized compounds **3-11** have significant antimicrobial activities, with **5, 6, 7, 9**, **10** and **11** having higher antimicrobial activities than that of the other prepared compounds. On the other hand, compounds **3, 5, 7, 9**, **10** and **11** exhibited interesting high antifungal activities, in addition to their antibacterial activities. Ciprofloxacin and ketaconazole were used as antibacterial and antifungal reference drugs, respectively.

**Table 2:** Antimicrobial activities of the new synthesized compounds **3-11**.

Compound No.	Inhibition zone in mm (at 50 µg/mL)			
	Gram positive bacteria		Gram negative bacteria	Fungi
	*Bacillus subtilis*	*S. aureus*	*Escherichia coli*	*C. albicans*
**1**	14	18	19	-ve
**3**	16	20	14	16
**4**	14	18	19	-ve
**5**	25	25	20	17
**6**	23	25	30	-ve
**7**	19	22	21	20
**8**	13	19	16	-ve
**9**	20	25	24	14
**10**	19	24	20	16
**11**	20	22	21	15
**Ciprofloxacin**	23	23	25	-ve
**Ketaconazole**	-ve	-ve	-ve	23

### 2.3. Structure-Activity Relationship (SAR)

From the results above (Table 2), we can conclude that pyridine and amide moieties are essential for antimicrobial activity. In the present work, we can suggest that the antimicrobial activity is due to:

- The presence of nitrogen heterocyclic rings.
- The presence of the amide linkage groups generally enhancing the activity.
- The difference in activity between the compounds which is due to the indicated subsistents in the used reagents of the molecule.

## 3. Experimental

### 3.1. General

Melting points (ºC) were measured in open glass capillaries using a Barnstead 9001 Electrothermal melting point apparatus and are uncorrected. NMR spectra were obtained on a Bruker AC 500 Ultra Shield NMR spectrometer (Bruker, Fällanden, Switzerland) operating at 500 MHz for ^1^H and 125 MHz for ^13^C; the chemical shifts are expressed in δ (ppm) downfield from tetramethylsilane (TMS) used as internal standard. Electrospray ionization mass spectra (ESI-MS) were recorded on a Waters QuatroMicro triple quadrupole tandem mass spectrometer at 4.0 and 3.5 kV for positive and negative ions, respectively. Elemental analyses (C, H, N, Cl, S) were in full agreement with the proposed structures within ± 0.4% of the theoretical values. Monitoring the reactions and checking the purity of the final products were carried out by thin layer chromatography (TLC) using silica gel precoated aluminum sheets (60 F254, Merck) and visualization with ultraviolet light (UV) at 365 and 254 nm.

### 3.2. Chemistry

#### 3.2.1. Synthesis of 2,6-bis-(methyl-D-alanylcarbonyl)pyridine carboxylate *(**3**)*

*Method A*: To a stirred cold mixture (-15 ºC) of 2,6-pyridine dicarboxylic acid (**1**, 0.167 g, 1 mmol) in cold dry tetrahydrofuran (100 mL) and ethyl chloroformate (0.216 g, 2 mmol), triethylamine (0.202 g, 2 mmol) was added, then after 10 min., D-alanyl methyl ester (0.206 g, 2 mmol) was added. The reaction mixture was stirred at -15 ºC for 3 h then for 12 h at r.t. The formed triethylamine hydrochloride was filtered off, and the solvent was evaporated under reduced pressure. The obtained residue was dissolved in 150 mL dichloromethane, washed with water, 1N hydrochloric acid, 1N sodium bicarbonate and finally with water and dried over anhydrous calcium chloride. Solvent was evaporated under reduced pressure to dryness and the obtained solid was crystallized to give **3**.

*Method B*: To a solution of D-alanyl methyl ester (0.206 g, 2 mmol), pyridine-2,6-dicarbonyl dichloride (**2**, 0.204 g, 1 mmol) in dichloromethane (15 mL) was added at -10 ºC with stirring. Triethylamine (0.2 mL, 2 mmol) was added dropwise to the reaction mixture with stirring in order to keep the reaction mixture slightly basic (pH ~ 8). Stirring was maintained for 3 hours at (-15 ºC) and 12 h at r.t.. The reaction mixture was then washed with water, 1N hydrochloric acid, 1N sodium bicarbonate and finally with water and dried over anhydrous calcium chloride. Solvent was evaporated under reduced pressure to dryness and the obtained solid was crystallized to give **3**. IR (KBr, cm^-1^): ν 3284 (NH), 1742 (C=O), 1680 (C=O). ^1^H-NMR (DMSO-d_6_): δ 1.56 (d, 6H, 2 CH_3_), 3.80 (s, 6H, 2OCH_3_), 4.70-4.81 (m, 2H, 2 CH), 8.05 (s, 2H, 2 NH exchangeable with D_2_O), 8.29-8.37 (m, 3H, pyrid-H). ^13^C-NMR: 18.20, 48.31, 52.60, 125.19, 138.93, 148.35, 162.89, 173.28. MS, *m/z* (%): 337 (M^+^, 4), 306 (24), 275 (100), 204 (36), 133 (18), 105 (65), 77 (86).

*N2,N6-Bis(1-hydrazinyl-1-oxopropan-2-yl)pyridine-2,6-dicarboxamide* (**4**). A mixture of **3** (0.24 g, 1 mmol) and hydrazine hydrate (0.8 mL, 16 mmol) in absolute ethanol (50 mL) was refluxed for 6 h. Excess solvent was evaporated under reduced pressure to dryness and the obtained residue was triturated with ethanol, the obtained solid was crystallized to give **4**. IR (KBr, cm^-1^): ν 3853-3280 (NH, NH_2_), 1661 (C=O). ^1^H-NMR (DMSO-d_6_): δ 1.44 (d, 6H, 2 CH_3_), 2.51 (s, 4H, 2NH_2,_ exchangeable with D_2_O), 4.52-4.55 (m, 2H, 2 CH), 8.15-8.21 (m, 3H, pyrid-H), 9.16 (d, 2H, 2 NH exchangeable with D_2_O), 9.27 (s, 2H, 2 NH exchangeable with D_2_O). ^13^C NMR: 17.90, 47.54, 124.52, 139.18, 148.74, 162.93, 171.19. MS, *m/z* (%): 339 (M^+^+2, 100), 306 (32), 275 (56), 235 (44), 133 (100), 105 (72), 102 (65).

*N2,N6-Bis(1-oxo-1-(2-(thiophen-2-ylmethylene)hydrazinyl)propan-2-yl)pyridine-2,6-dicarboxamide*** (5**). A solution of **4** (0.34 g, 1 mmol) and thiophene-2-carbaldehyde (0.224 g, 2 mmol) in absolute ethanol (100 mL) was refluxed for 6 h. The solvent was concentrated under reduced pressure, the obtained solid was filtered off, dried and crystallized to afford **5**. IR (KBr, cm^-1^): ν 3854-3676 (NH), 1669 (C=O). ^1^H-NMR (DMSO-d_6_): δ 1.50 (d, 6H, 2CH_3_), 4.56-4.57 (m, 2H, 2CH), 7.18, 7.19 (s, 2H, 2CH=N), 7.44-7.73 (m, 6H, Ar-H), 8.20-8.32 (m, 3H, pyrid-H), 9.14, 9.63 (2s, 4H, 4NH exchangeable with D_2_O). ^13^C NMR: 14.06, 16.68, 48.22, 60.62, 124.78, 125.03, 125.16, 128.01, 128.25, 128.81, 129.33, 130.97, 131.51, 133.76, 139.05, 139.41, 139.73, 145.52, 148.32, 155.77, 159.55, 163.10, 172.33. MS, *m/z* (%): 523 (M^+^-2, 2), 510 (4), 480 (5), 457 (3), 355 (8), 221 (100), 207 (12), 193 (15), 150 (45), 105 (18), 94 (5), 78 (15).

*N2,N6-Bis(1-(1,3-dioxoisoindolin-2-ylamino)-1-oxopropan-2-yl)-pyridine-2,6-dicarboxamide* (**6**). A suspension of **4** (0.34 g, 1 mmol) and phthalic anhydride (0.3 g, 2 mmol) in glacial acetic acid (50 mL) was refluxed for 3 h. The solvent was evaporated under reduced pressure, the residue was solidified with diethyl ester. The obtained solid was filtered off, dried and crystallized to give **6**. IR (KBr, cm^-1^): ν 3853-3675 (NH), 1661 (C=O). ^1^H-NMR (DMSO-d_6_): δ 1.60 (d, 6H, 2CH_3_), 3.36-3.50 (m, 2H, 2CH), 7.88-8.07 (m, 8H, Ar-H), 8.23-8.42 (m, 3H, pyrid-H), 9.81, 9.99 (2s, 4H, 4NH exchangeable with D_2_O). ^13^C NMR: 20.48, 47.18, 123.72, 124.05, 124.90, 129.40, 135.30, 135.32, 160.20, 167.92, 171.90. MS, *m/z* (%): 597 (M^+^, 0.16), 569 (12), 462 (5), 433 (4), 413 (8), 278 (6), 267 (6), 207 (45), 162 (55), 104 (100), 78 (18), 55 (20).

*N2,N6-Bis(1-(2-cyclohexylidenehydrazinyl)-1-oxopropan-2-yl)pyridine-2,6-dicarboxamide* (**7**). To a solution of cyclohexanone (0.196 g, 2 mmol) in absolute ethanol (50 mL) in the presence of few drops glacial acetic acid, compound **4** (0.34 g, 1 mmol) was added with stirring. The reaction mixture was heated under reflux for 6 h, and evaporated under reduced pressure to dryness. The obtained residue was solidified with n-hexane, the solid formed was filtered off, dried and crystallized to give **7**. IR (KBr, cm^-1^): ν 3268 (NH), 2934 (CH-aliphatic), 1664 (C=O). ^1^H-NMR (DMSO-d_6_): δ 1.56 (d, 6H, 2CH_3_), 1.62-1.65 (m, 6H, 3CH_2_, cyclohexyl ring), 2.28-2.30 (m, 4H, 2CH_2_, cyclohexyl ring), 3.66-3.76 (m, 2H, 2CH), 8.25-8.56 (m, 3H, pyrid-H), 8.96, 10.15 (2s, 4H, 4NH exchangeable with D_2_O). ^13^C NMR: 15.80, 23.85, 26.45, 35.10, 46.96, 124.58, 139.08, 148.14, 162.42, 163.65, 172.25. MS, *m/z* (%): 497 (M^+^, 0.1), 480 (0.1), 465 (0.2), 238 (16), 220 (100), 192 (55), 146 (35), 110 (62), 45 (54).

#### 3.2.2. Synthesis of N2,N6-Bis(1-(2-(1-(4-substituted-phenyl)ethylidene)hydrazinyl)-1-oxopropan-2-yl)pyridine-2,6-dicarboxamides **8** and **9**

A mixture of **4** (0.34 g, 1 mmol) and an acetophenone derivative, namely, *p*-methoxy- or *p*-nitro-acetophenenone (2 mmol) in absolute ethanol (100 mL) was refluxed for 6 h in the presence of glacial acetic acid (2 mL). The reaction mixture was concentrated under reduced pressure, the solid formed was collected by filtration, and purified by crystallization to give **8** and **9**. 

*N2,N6-Bis(1-(2-(1-(4-methoxyphenyl)ethylidene)hydrazinyl)-1-oxopropan-2-yl)pyridine-2,6-dicarbox-amide* (**8**). IR (KBr, cm^-1^): ν 3269 (NH), 2997 (CH-aliphatic), 1663 (C=O). ^1^H-NMR (DMSO-d_6_): δ 1.36-1.42 (m, 6H, 2CH_3_), 1.68 (s, 6H, 2CH_3_), 3.68 (s, 6H, 2OCH_3_), 4.22-4.28 (m, 2H, 2CH), 7.42-7.84 (m, 8H, Ar-H), 8.16-8.34 (m, 3H, pyrid-H), 9.34, 10.05 (2s, 4H, 4NH exchangeable with D_2_O). ^13^C NMR: 17.60, 18.15, 52.95, 56.84, 114.05, 126.12, 129.65, 124.80, 138.96, 149.55, 161.85, 162.32, 168.70, 177.80. MS, *m/z* (%): 601 (M^+^, 10), 552 (1), 512 (2), 252 (12), 235 (10), 221 (100), 193 (25), 178 (24), 150 (54), 78 (15).

*N2,N6-Bis(1-(2-(1-(4-nitrophenyl)ethylidene)hydrazinyl)-1-oxopropan-2-yl)pyridine-2,6-dicarbox-amide* (**9**). IR (KBr, cm^-1^): ν 3311 (NH), 1669 (C=O). ^1^H-NMR (DMSO-d_6_): δ 1.42-1.48 (m, 6H, 2CH_3_), 1.72 (s, 6H, 2CH_3_), 4.18-4.30 (m, 2H, 2CH), 7.24-7.85 (m, 8H, Ar-H), 8.22-8.42 (m, 3H, pyrid-H), 9.40, 10.15 (2s, 4H, 4NH exchangeable with D_2_O). ^13^C NMR: 17.45, 18.23, 52.88, 56.85, 114.35, 125.98, 129.48, 125.10, 138.92, 148.95, 162.05, 162.40, 168.65, 176.95. MS, *m/z* (%): 631 (M^+^, 8), 585 (4), 537 (6), 509 (12), 357 (8), 275 (100), 245 (35), 189 (62), 133 (46), 77 (48).

#### 3.2.3. Synthesis of macrocyclic octaamide tetraimides **10** and **11**

A suspension of **4** (0.34 g, 1 mmol) and 1,2,4,5-benzenetetracarboxylic acid dianhydride or 1,4,5,8-naphthalene tetracarboxylic acid dianhydride (1 mmol) in acetic acid (50 mL) was refluxed for 7 h. The obtained solid was collected by filtration, and crystallized to give **10** and **11**.

*Macrocyclic octaamide tetraimide*** 10**. IR (KBr, cm^-1^): ν 3853-3675 (NH), 1661-1668 (C=O). ^1^H-NMR (DMSO-d_6_): δ 1.42-1.72 (m, 12H, 4CH_3_), 3.54-3.68 (m, 4H, 4CH), 7.75 (s, 4H, Ar-H), 8.18-8.30 (m, 6H, 2 pyrid-H), 9.78, 10.05 (2s, 8H, 8NH exchangeable with D_2_O). ^13^C NMR: 18.95, 48.05, 123.88, 124.92, 129.52, 135.45, 136.10, 161.20, 168.92, 174.90. MS, *m/z* (%): 1038 (M^+^, 2), 1021 (4), 1006 (15), 237 (25), 182 (75), 162 (10), 112 (20), 92 (55), 59 (72), 43 (100).

*Macrocyclic octaamide tetraimide*** 11**. IR (KBr, cm^-1^): ν 3862-3578 (NH), 1665-1668 (C=O). ^1^H-NMR (DMSO-d_6_): δ 1.38-1.90 (m, 12H, 4CH_3_), 3.65-3.72 (m, 4H, 4CH), 8.05-7.75 (m, 8H, Ar-H), 8.23-8.35 (m, 6H, 2 pyrid-H), 9.85, 10.15 (2s, 8H, 8NH exchangeable with D_2_O). ^13^C NMR: 17.95, 51.15, 119.68, 123.45, 134.75, 138.23, 138.78, 148.75, 157.86, 160.05, 172.18. MS, *m/z* (%): 1138 (M^+^, 2), 1124 (4), 1067 (15), 863 (12), 569 (8), 295 (10), 275 (100), 204 (75), 133 (35), 59 (78).

## 4. Conclusions

A series of chiral linear and macrocyclic bridged pyridines has been prepared starting from pyridine-2,6-dicarbonyl dichloride (**2**). The structure assignments of the new compounds are based on chemical and spectroscopic evidence. The newly synthesized compounds **3-11** have been screened for their bactericidal and fungicidal activities. Among them compounds **5, 6, 7, 9**, **10** and **11** have antimicrobial activities higher than that of the other prepared compounds. On the other hand, compounds **3, 5, 7, 9**, **10** and **11** also exhibited interesting high antifungal activities in addition to their antibacterial activities. Ciprofloxacin and ketaconazole were used as antibacterial and antifungal reference drugs, respectively.
